# Pathology

**Published:** 1890-12-06

**Authors:** 


### THE LONDON POST-GRADUATE COURSE AT THE PADDINGTON INFIRMARY.

### PATHOLOGY.

### ON DR. KOCH'S CURE FOR CONSUMPTION.

Dr. Savill said that he was indebted for the following notes to his friend, Dr. Martin L. Brown, of Exeter, who had just returned from Berlin. The results of Dr. Brown's observations in Berlin he summarised under four heads: The phenomena produced by the inoculation of the fluid; suitable cases; the nature of the medium used; and conclusions.

1. *The Phenomena produced by the Injection.*—There is a latent period of six to eight hours, at the end of which time, if there be tubercle present in the individual, certain events may occur. To illustrate some of these the following cases were first quoted: A case of quiescent disease in the knee-joint, and who was the subject of lupus. He was inoculated for the lupus, and this started the knee-joint affection. Another who was inoculated turned out to be the subject of a tubercular meningitis and died. Another case

‡ The Hospital, Vol. 9, p. 43.

which was not known to be the subject of kidney mischief after inoculation developed symptoms of tubercular pyelitis, and bacilli appeared in the urine. Hence its great value as a means of diagnosis in uncertain cases. The reaction which sets in after six to eight hours is both local and constitutional. An acute hectic fever ensues, but no shivering, the temperature runs up to 105-106, with profuse sweating, and sometimes a rash appears, which may resemble that of measles or that of scarlet fever. With regard to the local signs, these are best seen in cases of lupus. An enormous amount of swelling in the regions of the deposit occurs, due to an exudation of serum, together with considerable congestion of the parts about. In the case of pulmonary phthisis, if there be dulness the area of dulness will be much increased in extent after inoculation; hence an area of one inch of dulness may be increased to two or three inches. Lungs with extensive tubercular deposit may become completely consolidated, sometimes with fatal result. There is also a great increase of expectoration, together with increase of the cough; in some cases this is also followed by dyspnœa. All of these symptoms may disappear in two or three days, if no fresh fluid be injected.

2. The following are *suitable cases;* all forms of lupus, especially so, as the local process can be watched. Strumous glands swell up enormously at first, and then subside. Strumous joint diseases, even Pott's disease of the vertebræ. A child who was the subject of hip-joint disease was inoculated with small doses and suffered great pain afterwards, but in a few days was up and running about the ward. With regard to the lungs the result depends upon the stage of the disease. These stages are artificially divided into three. Cases in the early stage, that is with slight consolidation, improve after two or three weeks. Cases of the second stage, with consolidation and early breaking down, require great care, as hæmoptysis may be set up. With regard to cases in the third stage, with cavities, it can be only hoped that these may be eventually benefited by the treatment. The signs by which early phthisis can be detected are now of very great importance to the physician. They may be divided into eight: 1, History of heredity; 2, Loss of weight with no obvious cause; 3, Hæmoptysis; 4, Impaired movement on one or both sides of the chest walls; 5, Impaired resonance in the supra-scapular or supra-clavicular regions; 6, Ausculatory signs, sometimes there may be only prolonged expiration, a dry crackle, or even crepitation or pleuritic friction at the apex; 7, Elevation of the temperature at night; 8, Bacilli in the sputum.

3. *The Nature of the Fluid Inoculated.*—It is pale brown and clear, somewhat like a weak solution of Liebig's extract of meat. There are two hypotheses concerning its nature: one is, that it is an attenuated tubercular virus; and the other, that it is a ptomaine, *i.e.*, an animal alkaloid. It is certainly not a mineral substance. With regard to the dose, one c.c. is taken of the fluid (a gram by weight, *i.e.*, 15·432 grains). Roughly, this is 15½ drops. There is added to this 9 c.c. of a ½ per cent. solution of carbolic acid in water—that is, a 10 per cent. solution is made. One c.c. of this is taken, and diluted with 10 times its bulk of water; a one per cent. solution is thus produced. For external lupus, &c., one starts with a centigram of this solution. For cases of lung affection, c.m.—*i.e*, 1½ drops—and the dose is gradually increased, being doubled each time until reaction is obtained. It is occasionally cumulative in its action. With respect to *clinical events*, deaths have occurred, and some of the supposed cures may be only temporary, and we must wait six months or a year before we can be certain of the ultimate result.

Dr. Savill also showed the following parasites under the microscope: Sarcinæ ventriculi, Trichophyton tonsurans, Acarus Scabiei, Ecchinococci hooklets, the eggs of Bilharzia hæmatobia, Pediculi pubis, vestimenti, and capitis.

